# Supplementary material for: Synthesis and the Crystal Structure of a New Chiral 1D Metal–Organic Coordination Polymer Based on L-Prolineamide-Substituted Diarylacetylenedicarboxylic Acid Derivative
Source: Molecules. 2022 Nov 30;27(23):8376. doi: 10.3390/molecules27238376 (PMC9738848; doi:10.3390/molecules27238376)
Supplement: Supplementary file 1 [file molecules-27-08376-s001.zip › molecules-2050312-supplementary.pdf]

Supporting Information

Synthesis and the Crystal Structure of a New Chiral 1D Metal-Organic Coordination Polymer based on L-Prolineamide-Substituted Diarylacetylenedicarboxylic Acid Derivative

Vladimir V. Veselovsky [1], Vera I. Isaeva [1], V. D. Nissenbaum [1], Leonid M. Kustov [1,2] and Vladimir V. Chernyshev [3,4,]*

[1] N.D. Zelinsky Institute of Organic Chemistry, Russian Academy of Sciences, Leninsky prospect 47, Moscow 119991, Russia
[2] National University of Science and Technology MISiS, Leninsky prospect 4, Moscow 119991, Russia
[3] Department of Chemistry, M.V. Lomonosov Moscow State University, 1-3 Leninskie Gory, Moscow 119991, Russia;
[4] A.N. Frumkin Institute of Physical Chemistry and Electrochemistry, Russian Academy of Sciences, Building 4, 31 Leninsky prospect, Moscow 119071, Russia
* Correspondence: vladimir@struct.chem.msu.ru (VVC)

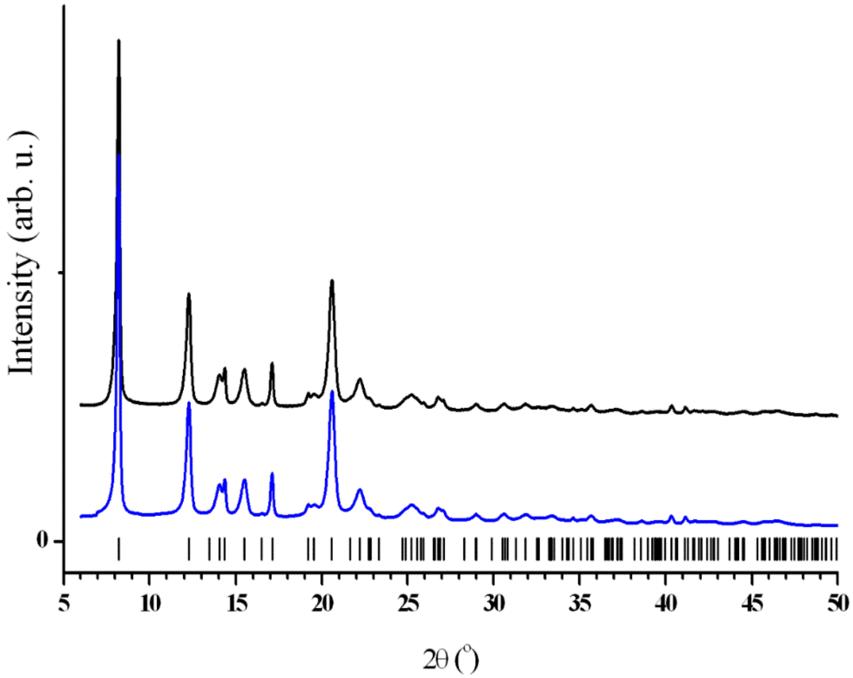

Figure S1. Comparison of experimental (top, in black) and calculated (bottom, in blue) powder patterns for [$\mathbf{Cu_2(EDPB)\bullet H_2O}$]$_\mathbf{n}$ in 6-50° 2θ range. The vertical black bars denote calculated positions of the diffraction peaks.

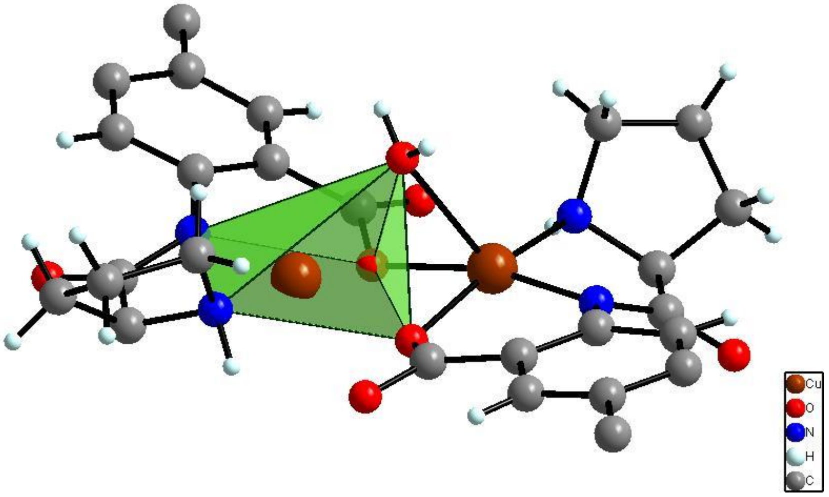

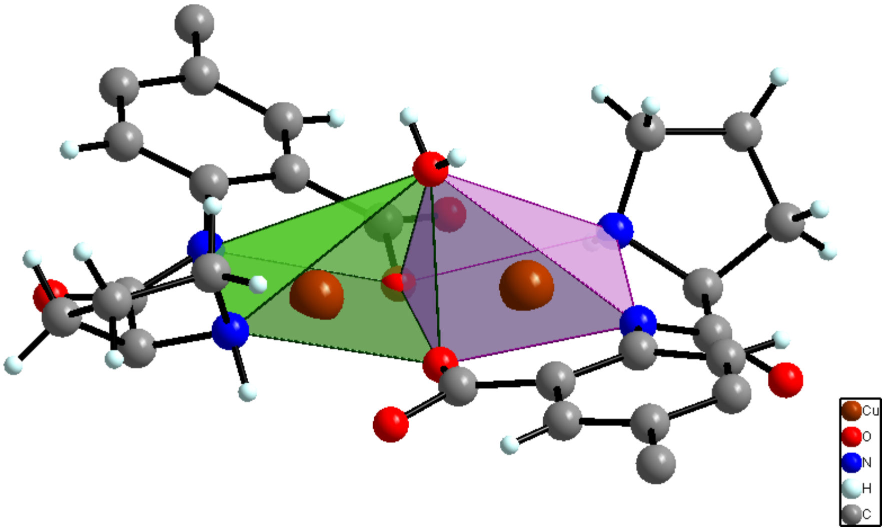

Figure S2 (prepared with DIAMOND [1]). A portion of the crystal structure of **[Cu_2(EDPB)•H_2O]$_n$** showing the heavily distorted square-base coordination pyramid (top), and two Cu-centered coordination polyhedra merged by sharing of a triangular face (bottom).

(1) Brandenburg, K. (1999). *DIAMOND*. Crystal Impact GbR, Bonn, Germany.

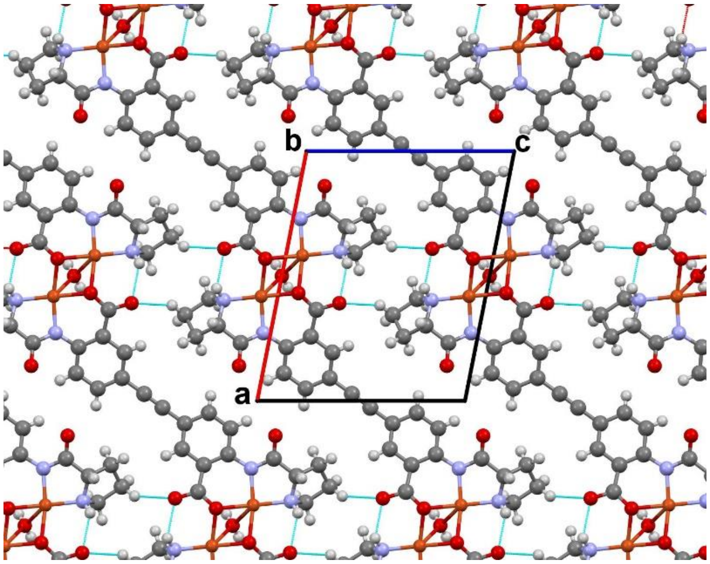

Figure S3. 2D layer in **[Cu_2(EDPB)•H_2O]$_n$** formed by N2–H2…O2^{i} and C11–H11A…O2^{iii} hydrogen bonds (see Table 2) viewed along the *b* axis. Hydrogen bonds are shown as thin blue lines.

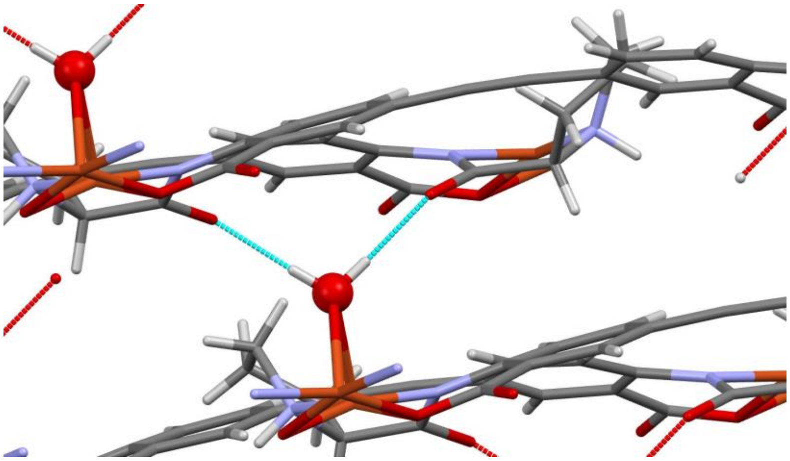

Figure S4. A portion of the crystal packing in **[Cu_2(EDPB)•H_2O]$_n$** showing intermolecular O4-H4…O3^{ii} (see Table 2) hydrogen bonds (thin blue and red lines), which link 1D polymeric chains from various 2D layers and consolidate the 3D structure.

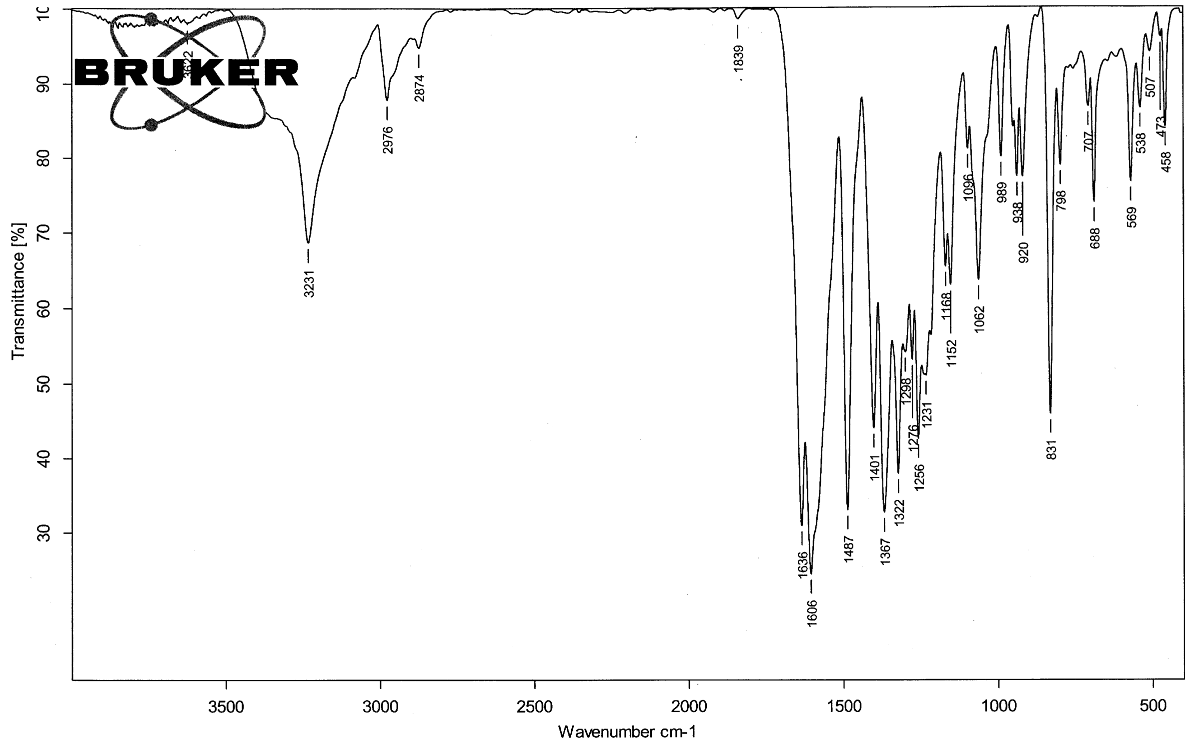

Figure S5. IR spectrum of the [**Cu_2(EDPB)•H_2O]$_n$** polymer.

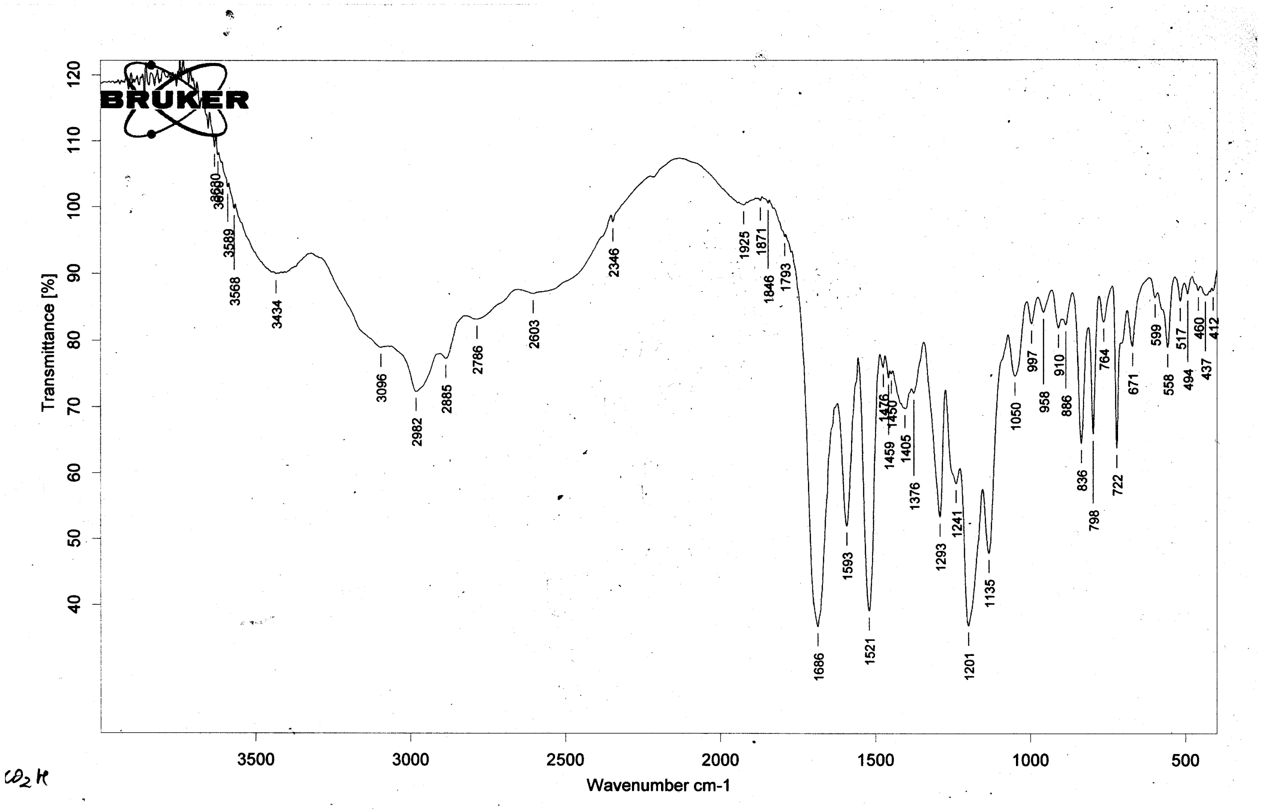

Figure S6. IR spectrum of the EDPBA linker.

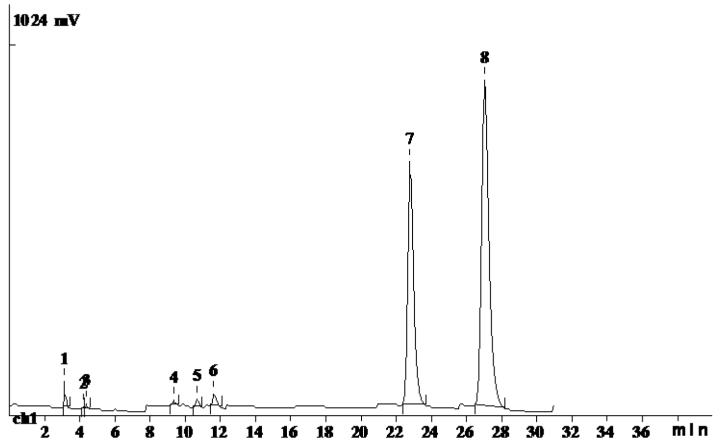

index	Time (min)	Area (mV/sec)	Area (%)
5	10.69	251.80	0.58
6	11.67	532.44	1.22
7	22.80	15612.13	35.92
8	27.03	26399.02	60.73
	31.06	~43400	~100

Figure S7. HPLC analysis of the product of 4-nitrobenzaldehyde and nitromethane Henry reaction.
